# Process of Fragment-Based Lead Discovery—A Perspective from NMR

**DOI:** 10.3390/molecules21070854

**Published:** 2016-07-16

**Authors:** Rongsheng Ma, Pengchao Wang, Jihui Wu, Ke Ruan

**Affiliations:** Hefei National Laboratory for Physical Science at the Microscale, School of Life Sciences, University of Science and Technology of China, Hefei 230027, Anhui, China; mrs1988@mail.ustc.edu.cn (R.M.); wpc1009@mail.ustc.edu.cn (P.W.); wujihui@ustc.edu.cn (J.W.)

**Keywords:** fragment based lead discovery, NMR spectroscopy, protein–protein interaction

## Abstract

Fragment-based lead discovery (FBLD) has proven fruitful during the past two decades for a variety of targets, even challenging protein–protein interaction (PPI) systems. Nuclear magnetic resonance (NMR) spectroscopy plays a vital role, from initial fragment-based screening to lead generation, because of its power to probe the intrinsically weak interactions between targets and low-molecular-weight fragments. Here, we review the NMR FBLD process from initial library construction to lead generation. We describe technical aspects regarding fragment library design, ligand- and protein-observed screening, and protein–ligand structure model generation. For weak binders, the initial hit-to-lead evolution can be guided by structural information retrieved from NMR spectroscopy, including chemical shift perturbation, transferred pseudocontact shifts, and paramagnetic relaxation enhancement. This perspective examines structure-guided optimization from weak fragment screening hits to potent leads for challenging PPI targets.

## 1. Introduction

As great things almost always start small, fragment-based lead discovery (FBLD) blossomed two decades after the first screening against a small set of low-molecular-weight compounds [1], then dubbed fragments. Since then, FBLD has gained increasing popularity in large pharmaceutical companies and biotechnology laboratories [2,3]. The anti-melanoma drug Vemurafenib represents the first approved drug using FBLD [4]. This year, Bcl-2 inhibitor Venetoclax, which originated from NMR screening [5], has been approved for the treatment of chronic lymphocytic leukemia [6]. Tens of drug candidates derived from FBLD are now in different phases of clinical trials [3,7]. Considering the 12- to 18-year time lag between the initial screening and final approval, we expect more fragment-derived drugs to benefit more patients.

As a complementary method to the widely-used high throughput screening (HTS) [8], FBLD also aims to discover micromolar affinity/activity leads. FBLD screens against low-molecular-weight compounds (normally less than 25 heavy atoms or 250–300 Da) instead of the lead-like compounds at approximately 500 Da that are used for HTS. Due to the low structural complexity of the fragments, the chemical space of drug-like compounds with 11 heavy atoms or fewer is ca. 10^9^ [9,10], versus the circa 10^60^ possible lead-like compounds for HTS [11]. Therefore, FBLD normally has a much higher hit rate than HTS, even if a small library (several hundred to thousand compounds) is recruited for FBLD in comparison with an approximately 1 million compound HTS library.

The small size of the compounds means a better chance of fully matching the interactions with targets [12]; i.e., high ligand efficiency that is defined as the binding energy per heavy atom [13,14]. Such intrinsically weak but high quality interactions between targets and fragment screening hits can be well-characterized by biophysical approaches such as surface plasmon resonance (SPR) [15] and nuclear magnetic resonance (NMR) [16,17,18]. Although X-ray crystallography provides the most detailed delineation of protein–ligand binding modes, its application in primary fragment-based screening has been limited so far [19]. To increase the occupancy levels of weak binders in protein pockets, a high concentration of fragments is necessary, which is often limited by the aqueous solubility of the fragments. NMR spectroscopy has played a vital role in screening since the naissance of FBLD [1] due to its power to characterize various properties of weakly-bound target-ligand complexes using multiple techniques [20,21,22]. NMR fragment-based screening identifies direct binders to the target protein. Conversely, HTS campaigns, which use cellular or biochemical assays to test a large collection of small molecules, suffer from a large number of false positive hits from promiscuous aggregators and pan-assay interference from compounds that undergo non-specific chemical reactions with proteins, such as redox compounds [23]. Fragment screening hits are further optimized to lead-like ligands, which is largely driven by detailed structural information from X-ray crystallography or NMR spectroscopy. In general, fragment-based screening samples a larger portion of chemical space, has a higher hit rate and ligand efficiency, and suffers less from false positive results when compared with HTS [3]. It is also intuitive for medicinal chemists to develop the fragment hits into potent leads.

Thanks to these merits, FBLD has been extensively applied to not only enzymes but also novel protein–protein interactions (PPIs) [24,25]. Targeting PPIs is considered the high hanging fruit in drug discovery [26]. Taking into account the complexity of the protein–protein interaction networks that underlie profound biology, it is of great interest to block specific protein–protein interactions to elucidate correlated phenotypes. However, the large, shallow, elongated, and flexible protein–protein interfaces pose grand challenges for conventional high-throughput screening campaigns [27,28]. For some PPIs, the binding energy is mainly contributed by a limited number of residues that form a “hot spot” [29]. This “hot spot” can accommodate small molecules identified from fragment-based screening, which are eventually evolved into potent leads.

In this perspective, we describe NMR fragment-based screening procedures, including fragment library construction, NMR ligand and protein observed screening spectra, and our technical concerns. NMR approaches, which provide valuable structural information at the initial hit-to-lead stages, are also delineated. These include chemical shift perturbation to map the binding site and the emerging techniques of transferred paramagnetic relaxation enhancement (PRE) and pseudocontact shifts (PCSs) that determine the distance and/or orientation of fragment hits with respect to target proteins. We examine the success of FBLD in the most recently published fragment-derived leads targeting challenging protein–protein interactions.

## 2. Prioritization of Targets

Aside from extensively-studied targets of enzymes and G-protein coupled receptors, novel PPI targets have gained increasing popularity in the last decade. The complex protein–protein interaction networks are heavily involved in many biological processes associated with diseases—including tumor growth, metastasis, and apoptosis [27,29]. For instance, Nutlin, which blocks the MDM2-p53 interactions to stabilize p53, has progressed to clinical trials [30]. Most recently, epigenetic targets have become a new frontier in drug discovery [31], including anti-cancer and anti-inflammation inhibitors that block the recognition of acetylated histones via bromodomains and induce profound chromatin biology [32,33,34,35,36,37]. Prior to the extensive research from initial screening to lead generation, how can we prioritize these targets and assess their druggability?

The traffic-light system has been introduced to prioritize potential drug targets based on the following five indices: (1) target validation, (2) assay feasibility, (3) druggability, (4) toxicity, and (5) structural information [38]. For a target with a high priority, it is preferred that genetic and chemical evidence demonstrate the following traits: (1) the target is essential for desired phenotypes; (2) an assay is ready in plate format and sufficient amounts of the protein can be supplied in time; (3) drug-like inhibitors (candidates within the target family) are known and the active site has been predetermined; (4) selective inhibition is possible; and (5) the target–ligand complex structure is solved at a high resolution. Scoring over these five items allows the prioritization of targets for lead discovery. A low score for a particular item does not mean termination of the project. It rather requires more resources, such as structure determination, which may be quickly available from research institutes. In general, the traffic-light system allows us to interrogate targets comprehensively and improves the success rates for drug discovery.

## 3. NMR Fragment Library

From a statistical point of view, it is more likely for fragments to match target binding motifs [12,39]. As the complexity of small molecules increases, some parts of the small molecules may weaken the protein–ligand interactions. The core idea is to design a library composed of low-molecular-weight compounds ranging from 150 Da to 300 Da. Similar to the well-known Lipinski’s rule of five (RO5) [40,41], the rule of three (RO3) has been proposed to guide the design of fragment libraries with the following tenets: (1) molecular weight ≤ 300 Da, (2) calculated logarithm of compound partition coefficient between n-octanol and water clogP ≤ 3, (3) hydrogen bond donors and acceptors each ≤ 3, (4) rotatable bonds ≤ 3, and (5) high aqueous solubility [42,43]. Many pharmaceutical companies and biotechnology laboratories have constructed their own fragment libraries with several hundred to several thousand compounds. Fragment libraries are either commercially available from vendors such as Maybridge and ChemBridge, or selected from millions of commercially available compounds and/or an internal library that satisfies the RO3.

If NMR is used as the main approach for fragment-based screening, special concerns should be addressed based on our experiences [44]. It is desirable for the fragments to have at least one aromatic proton to avoid strong interference from solvent protons, such as in HEPES buffer. Compounds without predicted signals in the area of interest are removed from the final library. For a concise and diverse library to cover the chemical space efficiently, compounds with high Tanimoto similarity scores to the current pool should also be filtered out. Such a process is not always mandatory, as we may intentionally deposit some analogues into the library to cross-validate the screening hits.

Quality control of these fragments is required prior to the first NMR fragment-based screening [44,45]. The fragments are stored in plate format at a concentration of approximately 200 mM in DMSO-*d*_6_, and then diluted to approximately 1 mM in aqueous buffer. The 1D proton spectra for all of the compounds are acquired one by one using an NMR spectrometer equipped with an autosampler. These 1D spectra serve multiple purposes. First, they measure the aqueous solubility of the compounds through comparison of the intensities per proton with standard samples. We recommend filtering out compounds with an aqueous solubility less than 0.1 mM or 0.2 mM, as high aqueous solubility is a prerequisite for screening, affinity, and structural studies. Second, these reference spectra are used to identify hits from future screening results. Third, they quantify the percentage of impurities and verify the proposed structures. Finally, these spectra allow for the preparation of cocktails with less signal overlap, which will be discussed in the following section.

## 4. NMR Methods for Fragment-Based Screening

Many biophysical approaches, including SPR [46,47], NMR [18,48], X-ray crystallography [49,50], isothermal titration calorimetry (ITC) [51], and microscale thermophoresis [52,53] have been applied in fragment-based lead discovery and biochemical high-content screening [54]. It is highly recommended to integrate as many techniques as possible into FBLD to remove distracting false positive results. Among these techniques, NMR has the unique power to detect weak protein–ligand interactions with multi-valued outputs [55]. Both ligand-observed and protein-observed spectra are extensively used in fragment-based screening.

### 4.1. Ligand-Observed NMR Fragment-Based Screening

The ligand-observed NMR screening spectra measure changes in the intensity, sign, and relaxation of the ligand signals in the presence of a small portion of protein. Saturation transfer difference (STD) [56,57] spectra are almost always used in NMR fragment-based screening. STD uses a long train of selective pulses to saturate protein methyl signals; the saturation diffuses throughout the protein and then transfers to binders to cause signal decay. Whether alternatively saturating the methyl signals or not, the difference spectra show signals of binders but not non-binders. WaterLOGSY detects the intermolecular water–ligand nuclear Overhauser effects (NOEs), whose signs are inverted in the ternary water–ligand–protein complex [22,58,59]. The Carr–Purcell–Meiboom–Gill sequence (CPMG) utilizes the line broadening effect of ligands upon binding to proteins with transverse relaxation rates determined in the absence and presence of proteins [60,61]. Other NMR techniques, including ^19^F-NMR [62,63] and diffusion experiments [64,65], have also have been used in fragment-based screening [66,67]. ^19^F-NMR is particularly useful for fluorine-containing inhibitors and is exemplified for binding and functional screening [68,69]. It is worth noting that artifacts exist for all screening spectra; for example, ligand methyl protons might be unwantedly saturated causing false positive STD signals, especially for aliphatic protons. However, for very hydrophobic binding pockets, we may fail to observe inverted WaterLOGSY signals for binders. Therefore, we recommend the acquisition of multiple NMR spectra if possible for cross-validation of screening hits.

Due to the dispersed chemical shifts in the ligand-observed NMR spectra, cocktails are usually used for approximately 10 compounds to significantly improve the throughput. Because the hit rates for primary screening are normally 0.1%–8% depending on the druggability of the targets [70], it is unlikely to have more than two hits in the same cocktail. Therefore, we compare the 1D reference spectra acquired for each individual fragment and pool those compounds with fewer overlapping pair-wised peaks into the same cocktail [44]. It is a laborious process to pipette fragments into cocktails. Hence, we recommend preparing cocktails in a sufficient amount for screening over many targets; for example, 20 mM for each compound in a stock solution of 100–200 μL of DMSO-*d*_6_. The cocktails are then diluted into the appropriate aqueous buffer. To avoid the strong WaterLOGSY signals observed in Tris buffer, a phosphate or HEPES buffer is preferred for ligand-observed NMR screening.

To calibrate the NMR parameters related to screening spectra, we recommend preparing a standard sample composed of 1 mM tryptophan (binder) and sucrose (non-binder) and 0.1 mM bovine serum albumin. STD parameters, including the saturation power, length (approximately 1 s) and frequency (normally at −1 to 0 ppm), can be well tuned using this sample to obtain maximum signal intensity for tryptophan without artificial signals from sucrose. Accordingly, the water-selective pulse in WaterLOGSY is also optimized to achieve strong signals for tryptophan, but inverted weak signals for sucrose.

**Figure 1:**
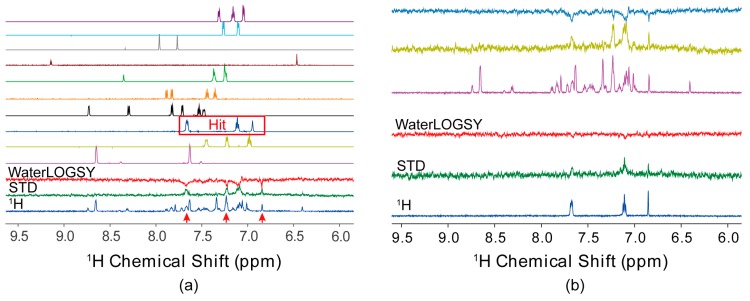
Ligand-observed NMR fragment-based screening. (**a**) The primary screening spectra for a typical cocktail. The top ten are the reference spectra for each component in the cocktail, while the bottom three are the WaterLOGSY, saturation transfer difference (STD), and ^1^H spectra for the cocktail as annotated. Red arrows denote signals from the hits. Slight chemical shift displacements may be observed because the reference and screening spectra were acquired in different buffers. (**b**) Secondary screening spectra in singleton for the identified hits. The top three spectra from the primary screening are overlaid with the secondary ones (bottom three) for comparison.

Once these NMR parameters are optimized, the primary screening against cocktails is carried out in a highly-automated way. The cocktails in DMSO-*d*_6_ are diluted to a final concentration between 0.2 mM and 1 mM in the aqueous buffer, depending on the biocompatibility in DMSO and spectral sensitivities. The protein concentration is set to 2–10 μM, which is often limited by the availability of the proteins. Certainly, higher concentrations of proteins and cocktails lead to higher sensitivity of NMR spectra. For an Agilent NMR spectrometer equipped with the 96-well autosampler, the single command “csv2cpQ” transfers user-defined parameters to the spectrometer, including the sample name, position, and pulse schemes. The NMR spectra are then acquired one-by-one with automatic shimming for each sample tube. The screening datasets are automatically stored in a shared network drive and processed/analyzed using third-party software (Figure 1a), such as ACD/Labs or Mnova [44,71]. In many cases, the buffer conditions are optimized to be compatible with proteins, which might differ from those used for the reference spectra. Therefore, the chemical shifts in the reference and primary screening spectra can be quite different. Therefore, manual inspection is necessary in most cases to identify hits from the crowded and low sensitivity screening spectra.

To validate the primary screening results, it is desirable to run a secondary screen for individual hits using the same experimental settings. Comparison of the primary and secondary screen spectra (Figure 1b) allows validation of the deconvolution process involved in the primary screen. The secondary screening also removes ambiguous hits due to the imperfect cancellation of signals in the STD and WaterLOGSY spectra or spectral instability. Using the aforementioned NMR screening settings, we have identified a series of hits for nine protein–protein interaction targets, with a primary screening hit rate of 0.7% ± 0.4%. Such a hit rate has also been used to evaluate the druggability of target proteins [70].

### 4.2. Protein-Observed NMR Fragment-Based Screening

**Figure 2:**
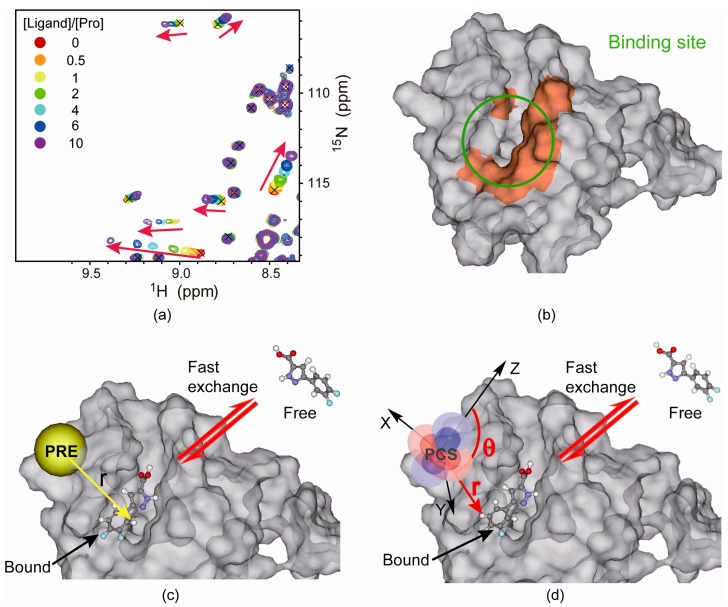
A structural model of a weakly bound protein–ligand complex derived from limited NMR restraints. (**a**) Chemical shift perturbations of the target protein detected by the ^1^H, ^15^N heteronuclear single quantum coherence (HSQC) spectra upon titration of a hit; (**b**) The binding epitope mapped by chemical shift perturbations; (**c**) Paramagnetic relaxation enhancement (PRE) provides distance restraints between the paramagnetic spin labels immobilized on the protein and the bound ligand; (**d**) The pseudocontact shifts provide both distance and orientation restraints between the protein-chelated paramagnetic lanthanide ion and the bound ligand.

Chemical shift perturbation experiments are the first method applied in FBLD [1], and this technique is probably the most widely used among all NMR approaches to study protein–ligand [72], protein–protein [73], and protein–nucleic acid [74] interactions. For small to medium sized proteins (generally less than 25 kDa), the ^1^H, ^15^N heteronuclear single quantum coherence (HSQC) spectra are acquired as fingerprints of amide chemical shifts for the target ^15^N-labeled protein (approximately 50 μM) upon the titration of excessive fragments. The chemical shift changes for each residue, Δδ*_obs_*, are defined with the following equation:

(1) $$\Delta \delta_{obs}=\sqrt{(\Delta \delta_{H}^{2}+{\left(\alpha \Delta \delta_{N}\right)}^{2})/2}$$

where Δδ*_H_* and Δδ*_N_* denote the chemical shift changes with respect to the *apo*-form protein along the ^1^H and ^15^N dimension in ppm, respectively. The constant *α* is normally set to 0.1 to 0.2 to balance the contributions from the ^1^H and ^15^N chemical shift changes. Statistically, the binding site residues are identified if their chemical shift changes are significantly deviated from the mean value. If spectrometer time is allowed, the chemical shift changes can be detected at a series of ligand/protein molar ratios, such as 0.0, 0.25, 0.5, 0.75, 1.0, 2.0, and 4.0 (Figure 2a). Due to the weak interactions between proteins and fragment screening hits, we normally observe fast exchanges upon ligand titration, which can be described with the following equation [72]:

(2) $$\Delta \delta_{obs}=\Delta \delta_{\max}\left\{\left({\left[P\right]}_{t}+{\left[L\right]}_{t}+K_{d}\right)-\sqrt{{\left({\left[P\right]}_{t}+{\left[L\right]}_{t}+K_{d}\right)}^{2}-4{\left[P\right]}_{t}{\left[L\right]}_{t}}\right\}/2{\left[P\right]}_{t}$$

where [*P*]_t_ and [L]_t_ denote the experimental protein and ligand concentrations, respectively. Δ*δ*_max_ represents the maximum residue-specific chemical shift change value, while *K*_d_ is the binding affinity common for all the residues. These parameters (Δδ_max_ and *K*_d_) are best-fitted from the correlation curve between chemical shift changes and ligand concentrations.

Chemical shift perturbation is probably the most robust method for fragment-based screening. Benefitting from the sensitivity enhancement by using a high magnetic field spectrometer equipped with a cryoprobe as well as the band-selective optimized flip-angle short-transient heteronuclear multiple quantum coherence (SOFAST-HMQC) pulse schemes [75], accessibility of spectrometer times may not be a major concern for protein-based screening. However, its application in primary fragment-based screening is still somewhat limited by the requirement of a large amount of ^15^N-labeled proteins (50 μM ^15^N-labeled proteins vs. 5–10 μM unlabeled proteins). To accelerate the primary screening process, the HSQC/HMQC spectra are normally acquired at one fixed ligand/protein molar ratio for a mixture of compounds. The hits are then identified from either titration of each individual component from the cocktail to the ^15^N-labeled protein or the ligand-observed spectra. For more details regarding protein-based fragment screening, please refer to the recent review by Harner et al. [18]. Taking these data into account, we prefer to use the ligand-observed spectra in the primary screening and to validate the screening hits using chemical shift perturbations if possible.

## 5. Generation of Structural Models from Sparse NMR Restraints

Once hits are identified, it is of paramount interest to elucidate the protein-hit binding modes to guide hit-to-lead evolution. It is always worthwhile to solve the complex structure using soaking or co-crystallization, which provide the most detailed delineation of binding modes. However, we may fail to solve the complex crystal structure due to low occupancy and/or low aqueous solubility of the weak binders. To solve such a bottleneck problem in fragment-based lead discovery, NMR spectroscopy is recruited as a highly complementary approach to provide structural restraints. Even sparse NMR restraints are very useful for the generation of an initial structural model, which can be refined based on further structure–activity relationship analysis.

The binding epitope can be retrieved from chemical shift perturbation experiments, assuming that the backbone chemical shift assignment is available *a priori* (Figure 2b). The binding residues are identified from the chemical shift perturbations upon titration of the fragment hits or natural substrates. For competitive fragment hits, a common set of residues are disturbed. The ^1^H, ^15^N HSQC is widely used to identify binding site residues, though ^1^H-^13^C HSQC/HMQC is also used but is less cost effective [76]. Assuming that the significantly perturbed residues are within a 4 Å distance from the fragment hit, a rough complex structural model can then be generated using docking programs such as AutoDock [77,78].

More quantitative distance restraints can be retrieved from the interligand nuclear Overhauser effects (ILOE), which are observed between a pair of compounds binding simultaneously in close proximity on the surface of a receptor protein [79]. Such protein-mediated ILOEs are experimentally observed using a 2D ^1^H-^1^H NOE spectrum for a ternary complex. This interligand distance restraint is adopted to guide the synthesis of bidentate inhibitors from the two individual hits [80,81,82]. It requires neither isotope labeling nor any structural information on the protein and is therefore applicable to even large proteins. It is noteworthy that the ILOE results should be interpreted with caution to eliminate possible artifacts from transfer NOEs [83]; for example, the protein-relayed NOEs might also be observed if two hits bind sequentially to the protein.

NMR paramagnetic relaxation enhancement (PRE), which has gained extensive applications in protein structure and dynamics studies [84,85,86], has been recently used to quantitatively probe protein–ligand interactions [87]. Proteins are first modified with site-directed mutagenesis to generate a single solvent-accessible cysteine residue, which is then chemically linked via a disulfide bond to a paramagnetic center, such as the free radical of nitroxide or paramagnetic metal ions such as Cu^2+^, Mn^2+^, or Gd^3+^. The lone pair electrons from the paramagnetic center induce distance-dependent signal decay, which is also known as the PRE effect. Upon binding to spin-labeled proteins, the signals from the fragment hits also decay. Such an effect is quantified as the difference in the transverse relaxation rates under paramagnetic and diamagnetic conditions. The PRE effect is proportional to *r*^−6^, where r denotes the distance between the paramagnetic center and the atom of interest (Figure 2c). The paramagnetic center can be labeled at various sites to measure multiple PRE datasets. These restraints are then used to validate the binding modes from molecular docking.

Transferred pseudocontact shift is another emerging NMR approach to provide both orientation and distance restraints of ligands relative to proteins [88,89,90,91]. The targeting protein is mutated to have a single solvent-exposed cysteine. A lanthanide-binding tag (LBT) is then site-specifically labeled to the designated cysteine residue. The lanthanide chelating site should be selected with caution so that the LBT remains rigid relative to the protein and deviates from the protein–ligand binding site at an appropriate distance, such as 5–20 Å. Such a distance will allow a sensitive PCS measurement without too much signal decay due to the PRE effect. Upon binding to paramagnetic lanthanide ions with anisotropic chemical shift tensors, we observe chemical shift displacements for the nuclei of interest with respect to those in a diamagnetic condition, which are the so-called PCSs. PCSs describe the orientation and long-range distances (up to 50 Å) for nuclei of interest with respect to the paramagnetic center. Similar to the transferred PRE effect, the transferred PCSs [91,92] are observed for ligands upon binding to a small portion of proteins that are chelating paramagnetic lanthanide ions (Figure 2d). For small-to-medium sized proteins, protein PCSs may also be measured to determine the position of the paramagnetic center and the associated anisotropic chemical shift tensor. It is worth noting that bound-state PCSs are calculated assuming a fast exchange between the free and bound form of the ligand. Therefore, the observed PCSs are the population-weighted linear average over the free and bound states. The free-state PCSs of the ligand are zero, and the bound-state PCSs are then scaled according to the bound fraction. Such transferred PCSs are back-calculated from a proposed structural model, which can be refined until the experimental and calculated PCSs converge. Although a single bound-form conformation is assumed for most cases, the sparse restraints cannot exclude the possibility of multiple binding poses, especially for weakly bound protein–ligand complexes. Better structural models can be generated by labeling lanthanide ions at different protein positions, which act as a “GPS satellite system” to monitor the ligand distance and orientation with respect to the paramagnetic centers.

## 6. Structure-Guided Hit-to-Lead Evolution

Structure-guided hit-to-lead evolution has been extensively reviewed elsewhere [93,94]. In general, fragment growth, linking, and merging strategies have been used to evolve weak μM to mM affinity hits to nM potent inhibitors [95]. We refer the interested reader to a recent review for more examples regarding protein–protein interaction targets probed by NMR spectroscopy [96]. Here, we briefly introduce the application of NMR techniques in the discovery of epigenetic inhibitors to block the recognition of histone lysine acetylation via bromodomains. Inspired by the profound biology incurred by pioneering inhibitors for the bromodomain and extra-terminal (BET) family [32,33], where NMR STD, WaterLOGSY and CSPs have been recruited to delineate protein–ligand interactions from screening to lead generation [97,98,99], a significant amount of effort has been put into the discovery of new inhibitors of non-BET family bromodomains. Success in the identification of inhibitors targeting the low-druggability BAZ2B bromodomain using ligand-observed NMR fragment screening further ignited the passion for this campaign. We examine this optimization process from a recent case study of the epigenetic inhibitors targeting the ATAD2 bromodomain. The ATAD2 bromodomain has been considered very difficult to target from SiteMap analysis [100]. Fragment-based screening of ATAD2 bromodomain has hence been carried out independently by two groups using NMR and X-ray crystallography [101,102]. A series of hits has been identified with affinities of 350 μM or weaker, as determined by NMR chemical shift perturbation experiments.

**Figure 3:**
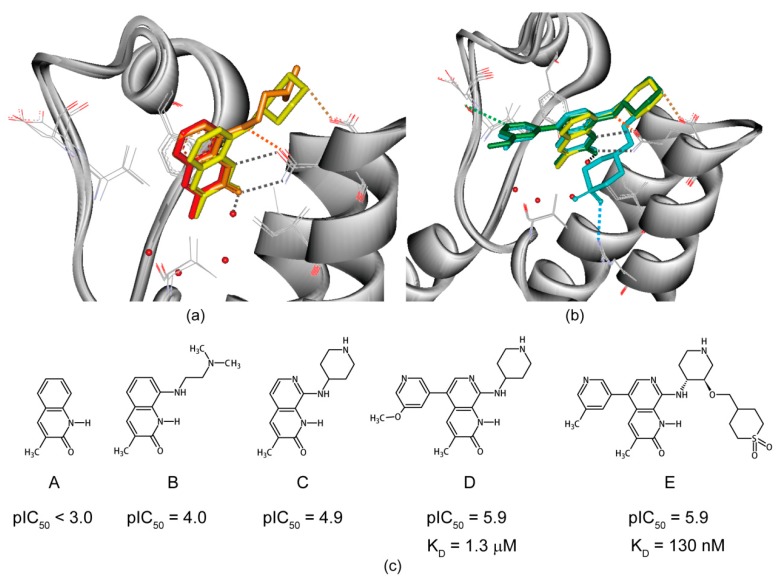
Hit-to-lead evolution in the discovery of ATAD2 bromodomain inhibitors. (**a**) Superimposition of the ATAD2 bromodomains in complex with fragment screening hits and derived analogues; (**b**) Structure-guided evolution from a micromolar affinity led to a nanomolar potent inhibitor; (**c**) Chemical structures of ATAD2 bromodomain inhibitors with their activities annotated. Compounds A–E are colored in red, orange, yellow, green, and cyan in (**a**,**b**), with PDB codes **5A5O**, **5A5P**, **5A5Q**, **5A5R** and **5A83**, respectively. The pIC_50_ and K_D_ values are measured using TR-FRET (time-resolved fluorescence resonance energy transfer) and SPR (surface plasmon resonance), respectively [103,104].

The protein-hit complex structure demonstrates that the hit warhead mimics acetyllysine (Figure 3). Because of the limited size of the acetyllysine binding pocket, the fragment growth strategy is used to introduce functional groups to the initial hit one-by-one. The complex crystal structures demonstrate that the designed functional groups interact with residues in the groove through hydrogen bonding and Van der Waals interactions (Figure 3) [103]. Selectivity over the bromodomain from the BET family protein BRD4 is monitored simultaneously. A potent and selective ATAD2 bromodomain inhibitor was eventually optimized from an initial 600 μM fragment screening hit, where complex structural studies played a key role [104].

## 7. Summary and Outlook

Fragment-based lead discovery is now entering its adulthood and has been proven fruitful for novel and challenging protein–protein interaction targets. The maturity of biophysical approaches such as NMR, X-ray crystallography, SPR, ITC and so on provide us a hawk-eyed view of weak protein–ligand interactions. Although NMR fragment-based lead discovery is the focus of this perspective, embracing all possible techniques is clearly the most effective way to develop potent and selective drug candidates. Further technical advances, especially X-ray crystallography and NMR spectroscopy, shall pave a highly automated way for the elucidation of exquisite protein–ligand interactions to guide hit-to-lead evolution and further our understanding of protein–protein interactions.

Clearly, fragment-based design will be a continued emphasis. More challenging targets involving allostery and conformational ensembles will also become druggable using FBLD, where excited-state conformations have now been better-characterized by various NMR techniques, such as paramagnetic relaxation enhancement, relaxation dispersion, chemical exchange saturation transfer, and ZZ exchange spectroscopy. Popular target classes will include epigenetic proteins, signal transduction networks, metabolic enzymes, etc. For example, bromodomains as histone lysine acetylation readers shall gain increasing attention in the near future, while histone methylation writers and erasers are emerging as druggable targets. As an emerging hallmark of cancer, the deregulation of cellular energetics is dictated by key metabolic enzymes, which could be of great therapeutic interest. Faster discovery of small molecule inhibitors will be boosted by close collaborations among experts in disciplines covering medicinal chemistry, chemical biology, structural biology, and cell biology. Importantly, more fragment-derived drug candidates will soon become drugs to benefit patients. Starting from small, FBLD makes many challenging targets druggable, including protein–protein interactions, which have been hailed as highly hanging fruits in drug discovery.
